# Association of angiotensin-converting enzyme gene insertion/deletion polymorphism (rs4646994) with schizophrenia in an eastern Algerian population: a case-control study

**DOI:** 10.11604/pamj.2025.52.65.46700

**Published:** 2025-10-09

**Authors:** Yasmina Boukhenaf, Ouarda Sariyah Ayachi, Amina Iness Bernou, Rayene Achou, Mohamed Lebsir, Fatima Zohra Madoui, Karima Sifi, Mohamed Larbi Rezgoun

**Affiliations:** 1 Laboratory of Molecular and Cellular Biology, Department of Animal Biology, University of Constantine 1, Constantine, Algeria; 2 Pathology, Diagnosis and Therapy, Health and Biotechnology Division, National Research Center of Biotechnology, Constantine, Algeria; 3 Psychiatric Hospital Mahmoud Belamri, Constantine, Algeria; 4 Laboratory of Biology and Molecular Genetics, University Hospital Center Benbadis, University of Constantine 3, Constantine, Algeria

**Keywords:** Angiotensin-converting enzyme, polymorphism, schizophrenia, genetics, case-control studies

## Abstract

**Introduction:**

the Angiotensin-Converting Enzyme (ACE) is a critical enzyme in the renin-angiotensin system and influences neurotransmitter regulation, including dopamine. Previous studies have suggested a potential association between ACE gene polymorphisms and psychiatric disorders, including schizophrenia. This study aimed to investigate the association between the insertion/deletion (I/D) polymorphism in the ACE gene and schizophrenia in an Eastern Algerian population.

**Methods:**

a case-control study was conducted involving 157 schizophrenia patients and 222 healthy controls. Deoxyribonucleic acid (DNA) was extracted from peripheral blood using the salting-out method, and the ACE I/D polymorphism was genotyped using the polymerase chain reaction (PCR) technique. Statistical analyses were performed using SPSS version 26.0.

**Results:**

no significant association was observed between the ACE I/D polymorphism and schizophrenia. Compared to the DD genotype, the ID genotype showed OR = 0.84 (95% CI: 0.47-1.50, P = 0.66), and the II genotype OR = 0.47 (95% CI: 0.05-4.54, P = 0.66). The I allele was also not significantly associated (OR = 0.80, 95% CI: 0.47-1.33, P = 0.38). Dominant, over-dominant, and recessive models showed no significant results.

**Conclusion:**

these findings suggest that the ACE I/D polymorphism does not contribute to the genetic susceptibility to schizophrenia in this population. Further research with larger sample sizes and consideration of gene-environment interactions is needed to provide deeper insights into the role of ACE in schizophrenia.

## Introduction

Schizophrenia (SCZ) is a chronic psychotic disorder affecting less than one percent of the general population. It is characterized by positive symptoms such as hallucinations and delusions, negative symptoms including social withdrawal and blunted affect, and frequent cognitive impairments in attention and memory [1]. Although its pathoetiology is not fully understood, SCZ is widely recognized as a polygenic and multifactorial disorder. Multiple association studies, including case-control designs, have highlighted the important contribution of genetic factors to disease susceptibility [2,3].

Among the numerous candidate genes investigated, the angiotensin-converting enzyme (ACE) gene has drawn attention because of observed alterations in ACE activity in various brain regions of patients with SCZ [4-7]. ACE is a key component of the renin-angiotensin system, catalyzing the conversion of angiotensin I to angiotensin II, degrading neuropeptides such as substance P, and modulating dopamine turnover in the midbrain [8,9]. Elevated ACE levels have been reported in the cerebrospinal fluid (CSF) of patients with SCZ, suggesting a possible link with neuroinflammatory processes in the disorder [10,11].

The ACE gene, located on chromosome 17q23, contains a common insertion/deletion (I/D) polymorphism in intron 16, defined by the presence or absence of a 287 bp fragment, which significantly affects ACE activity [8,9]. Numerous studies in different populations have examined the association between the ACE I/D polymorphism and SCZ. Results, however, have been inconsistent: some found no significant association [12-18], whereas others reported a link between the I allele and SCZ in Spanish patients [19] or between the D allele and SCZ in Turkish patients [20].

These discrepancies may reflect ethnic differences in allele distribution, environmental interactions, or sample size variations, and highlight the need for population-specific studies. In Algeria and more broadly in North Africa, no published research has investigated this association, despite the region´s distinct genetic background.

This study aimed to investigate the association between the ACE I/D polymorphism and SCZ in an Eastern Algerian population, thereby providing novel data from North Africa and contributing to the broader understanding of the genetic architecture of SCZ.

## Methods

**Study design and setting:** this case-control study was conducted in Constantine, Algeria. Patients with SCZ were recruited between January 2 and June 30, 2021, from the Mahmoud Belamri Psychiatric Hospital of Constantine, while control subjects were selected until December 2021 from medical personnel and healthy blood donors recruited at the Blood Transfusion Center of Dr. Benbadis University Hospital Center.

**Participants:** the schizophrenia group included 157 patients (121 males, 36 females) diagnosed according to the Diagnosis and Statistical Manual of Mental Disorders (DSM-V) criteria [21], through clinical interviews conducted by psychiatrists. Patients with other psychiatric disorders or intellectual disabilities were excluded. The control group consisted of 222 healthy individuals (180 males, 42 females) with no personal or family history of major psychiatric disorders or psychotic medication use.

**Variables:** the study investigated the association between the ACE I/D polymorphism and SCZ as the primary outcome. Independent variables included age, sex, and genotype distribution, while clinical variables were evaluated only in patients and comprised age of onset, Positive and Negative Syndrome Scale (PANSS) scores [22], and symptom severity.

**Data sources and measurement:** genomic DNA was extracted from 8 ml of venous blood collected into ethylenediaminetetraacetic acid (EDTA)-containing tubes using the salting out method [23]. The quality of the DNA was verified with a Nanodrop spectrophotometer (Thermo Scientific®, NANODROP 8000). Genotyping of ACE polymorphism (rs4646994) was performed by the polymerase chain reaction (PCR) method using specific primers: 5´-CTGGAGACCACTCCCATCCTTTCT-3´ (sense) and 5´-GATGTGGCCATCACATTCGTCAGAT-3´ (antisense). PCR reactions (25 µl) contained 50 ng genomic DNA, 0.2 µM of each primer, 1x PCR buffer, 0.2 mM dNTP, 1.5 mM MgCl_2_, and 0.5 U Taq DNA polymerase (Solis-Biodyne®). Cycling conditions were set as follows: 94°C for 5 min, 35 cycles of 94°C for 30 s, 56°C for 45 s, and 72°C for 30 s, and a final extension step of 72°C for 7 min. PCR products were separated on 3% agarose gels (Sigma®) stained with GelRed (Merck®) and visualized with the Gel Doc XR+ Gel Documentation System (Bio-Rad®). The insertion allele (I) appeared as a 490 bp band, and the deletion allele (D) as a 190 bp fragment.

**Bias:** to minimize potential sources of bias, cases and controls were recruited from the same geographic region to reduce population stratification bias. Genotyping was performed in a blinded manner regarding patient/control status. Additionally, statistical models were adjusted for age and sex to control for potential confounding factors.

**Study size:** the study included 157 schizophrenia patients and 222 controls, totaling 379 participants. The sample size was determined based on previous studies on ACE I/D polymorphism in schizophrenia, ensuring adequate statistical power for detecting potential associations while maintaining feasibility in recruitment and genotyping.

**Quantitative variables:** continuous variables were assessed for normality before analysis using the Shapiro-Wilk test. Normally distributed data were analyzed using ANOVA for comparisons between multiple groups, while non-normally distributed data were evaluated using the Mann-Whitney U test for two-group comparisons and the Kruskal-Wallis test for multiple-group comparisons. To preserve statistical power and avoid information loss, variables were analyzed in their continuous form whenever possible, without arbitrary categorization.

**Statistical methods:** the Chi-square goodness-of-fit test was used to evaluate the Hardy-Weinberg equilibrium. Allele and genotype frequencies were calculated and compared between the patients and control subjects using the standard chi-square test. Binary logistic regression analysis was performed to assess the association between genetic polymorphisms and SCZ, adjusting for covariates such as age and sex. The effect of different alleles and genotypes was estimated using Odds ratios (OR) and 95% confidence intervals (95% CI). Clinical associations, including PANSS scores, were analyzed across genotypes using ANOVA for normally distributed data and non-parametric tests for non-normally distributed data. Statistical significance was set at P < 0.05, and all analyses were performed using SPSS® version 26.0 (SPSS Inc., Chicago, USA).

**Ethical consideration:** written informed consent was obtained from all participants before collecting whole blood samples; all procedures performed in this study were in accordance with the Declaration of Helsinki (1964). The ethics committee of the Dr. Benbadis University Hospital Center of Constantine has approved this study; Reference Number: CE/CHUC/05/11-2023.

## Results

**Participants:** a total of 160 schizophrenia patients and 247 control subjects initially met the eligibility criteria and provided informed consent. However, after excluding samples due to DNA degradation or unsuccessful PCR amplification, the final study population consisted of 157 patients and 222 controls. Eligibility criteria included a confirmed diagnosis of SCZ based on DSM-V criteria for cases and the absence of any psychiatric disorder for controls. Individuals with a history of major neurological disorders, substance dependence, or severe medical conditions were excluded.

**Demographic and clinical characteristics:** the mean age of schizophrenia patients was 40.42 ± 10.51 years, while that of the control group was 38.65 ± 11.35 years. There was no significant difference in age between the two groups (P = 0.07). The gender distribution showed a predominance of males in both groups. For the patient group, 77.07% were male (n = 121) with a sex ratio (M/F) of 3.36. Similarly, the control group had a male frequency of 81.08% (n = 180), with a sex ratio (M/F) of 4.29. This difference was not statistically significant (OR = 0.78, 95% CI: 0.48-1.29, P = 0.34). Regarding smoking habits, patients exhibited a significantly higher smoking frequency compared to controls (58.66% vs. 33.01%; OR = 2.88, 95% CI: 1.87-4.45, P < 0.001). In addition, clinical evaluations of patients revealed mean PANSS scores of 31.26 ± 9.25 for positive symptoms and 23.11 ± 8.92 for negative symptoms.

**Figure 1:**
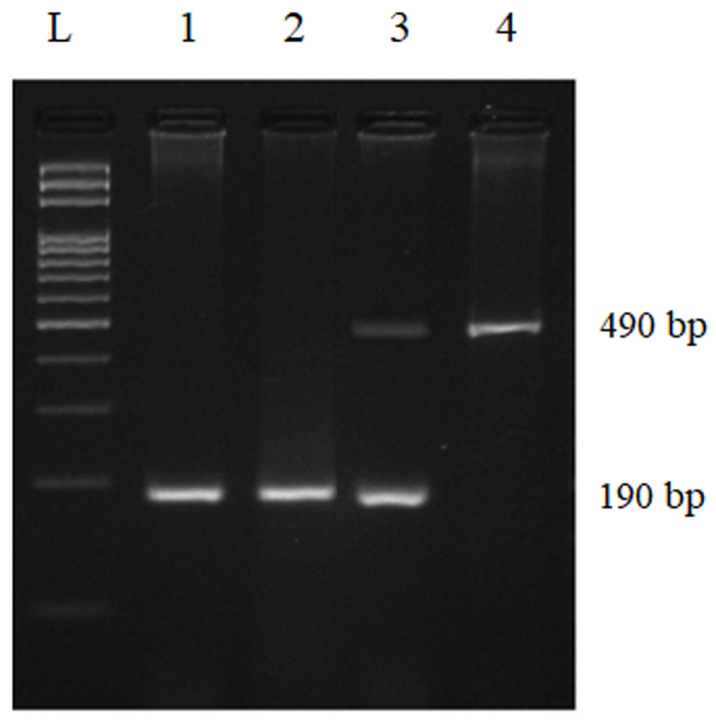
agarose gel electrophoresis of the polymerase chain reaction product of the angiotensin-converting enzyme insertion/deletion polymorphism (lane L: 100 bp ladder; lanes 1 and 2 are homozygotes for the D allele; lane 3 is a heterozygote for the I and D alleles; lane 4 is a homozygote for the I allele)

**Hardy-Weinberg equilibrium and genotype/allele frequency comparisons:** regarding the genotyping of the ACE I/D polymorphism, the results are given in Figure 1. Both populations were in Hardy-Weinberg equilibrium (cases χ^2^= 0.009, df = 1, P = 0.93; controls χ^2^= 0.63, df = 1, P = 0.43). The ACE I/D genotype and allele frequencies are shown in Table 1. The DD genotype was the most frequent in both patients (85.35%) and controls (82.43%). Using DD as the reference, we found that neither the ID genotype (OR = 0.84, 95% CI: 0.47-1.50; P = 0.66) nor the II genotype (OR = 0.47, 95% CI: 0.05-4.54; P = 0.66) was significantly associated with SCZ. Regarding allele frequencies, the D allele predominated in both patients (92.36%) and controls (90.54%). The I allele was slightly more frequent in controls, but the difference was not statistically significant (OR = 0.80, 95% CI: 0.47-1.33; P = 0.38).

**Table 1:** association of angiotensin-converting enzyme insertion/deletion polymorphism with schizophrenia: genotype and allele frequencies in patients and controls

Polymorphism I/D of ACE	Patients N (%)	Controls N (%)	P	OR (95 % CI)	P (adjusted)	OR (95 % CI) (adjusted)
**Genotypes**						
DD	134 (85.35)	183 (82.43)	0.66	1.00	0.64	1.00
ID	22 (14.01)	36 (16.36)		0.84 (0.47-1.50)		0.80 (0.45-1.44)
II	1 (0.64)	3 (1.36)		0.47 (0.05-4.54)		0.51 (0.05-5.02)
**Alleles**						
D	290 (92.36)	402 (90.54)	0.38	1.00	-	-
I	24 (7.64)	42 (9.46)		0.80 (0.47-1.33)		
**Genetic models**						
**Dominant**						
DD	134 (85.35)	183 (82.43)	0.45	1.00	0.39	1.00
ID+II	23 (14.65)	39 (17.57)		0.81 (0.46-1.41)		0.78 (0.44-1.38)
**Recessive**						
ID+DD	156 (99.36)	219 (98.65)	0.50	1.00	0.56	1.00
II	1 (0.64)	3 (1.35)		0.47 (0.05-4.54)		0.52 (0.05-5.16)
**Over-dominant**						
DD+II	135 (85.99)	186 (83.79)	0.56	1.00	0.47	1.00
ID	22 (14.01)	36 (16.21)		0.84 (0.47-1.50)		0.81 (0.45-1.45)

N: number; P: statistical significance (p-value); SCZ: schizophrenia; OR: odds ratio; CI: confidence interval; I/D: insertion/deletion

**Association of ACE I/D polymorphism with schizophrenia risk:** when evaluated under different genetic models, including dominant (ID+II vs. DD), recessive (II vs. ID+DD), and over-dominant (ID vs. DD+II) models, no significant associations were found between the ACE I/D polymorphism and SCZ. In the dominant model, the ID+II genotypes were not associated with SCZ compared to DD (OR = 0.81, 95% CI: 0.46-1.41; P = 0.45). Similarly, in the recessive model, the II genotype showed no significant effect when compared to ID+DD (OR = 0.47, 95% CI: 0.05-4.54; P = 0.50). For the over-dominant model, no association was found between the ID genotype and SCZ compared to DD+II (OR = 0.84, 95% CI: 0.47-1.50; P = 0.56). Logistic regression analyses adjusted for age and sex yielded consistent results, confirming the absence of a significant association between the ACE I/D polymorphism and SCZ across all genetic models.

**Angiotensin-converting enzyme I/D genotype and clinical variables in patients:** to explore potential associations between the ACE I/D polymorphism and clinical or demographic characteristics, additional analyses were performed (Table 2). Due to the low frequency of the II genotype (n = 1), the II and ID genotypes were grouped together (II + ID) to enhance statistical power and minimize biases. No significant associations were observed between genotype distribution and gender (OR = 0.92, 95% CI: 0.32-2.69, P = 0.88) or between genotype and age of onset (mean ± SD: 24.66 ± 6.88 years for DD vs. 23.71 ± 7.31 years for ID+II; P = 0.88).

**Table 2:** demographic and clinical variables divided according to the angiotensin-converting enzyme insertion/deletion polymorphism genotypes

Demographic features	Genotype DD	Genotypes (ID+II)	P
Gender (M/F)	103/31	18/5	0.88
Age of onset (years)	24.66 (6.88)	23.71 (7.31)	0.88
**PANSS score**			
Positive symptoms (mean (SD))	32.17 (8.78)	28.14 (10.45)	0.15
Hallucination (mean (SD))	5.19 (1.39)	4.93 (1.77)	0.72
Delusion (mean (SD))	5.25 (1.73)	4.29 (2.09)	0.13
Conceptual disorganization (mean (SD))	4.71 (1.74)	4.31 (1.70)	0.42
Negative symptoms mean (SD)	22.25 (8.68)	26.43 (9.46)	0.13
Passive/apathetic social withdrawal mean (SD)	2.89 (1.78)	3.71 (1.63)	0.07
General psychopathology (mean (SD))	45.37 (10.50)	43.07 (10.87)	0.47
Total score (mean (SD))	98.69 (19.07)	100.47 (22.49)	0.76
Smoking (smokers/non-smokers)	73/54	15/8	0.49
Cannabis (users/non-users)	15/112	2/21	0.67

PANSS: positive and negative syndrome scale

Regarding clinical variables, although no statistically significant associations were found between genotypes and PANSS scores, patients with the DD genotype showed higher mean scores for positive symptoms overall (32.17 ± 8.78) compared to those with ID + II genotypes (28.14 ± 10.45; P = 0.15). This trend was also observed for individual positive symptoms, including hallucinations (5.19 ± 1.39 for DD vs. 4.93 ± 1.77 for ID + II; P = 0.72), delusions (5.25 ± 1.73 vs. 4.29 ± 2.09; P = 0.13), and conceptual disorganization (4.71 ± 1.74 vs. 4.31 ± 1.70; P = 0.42). Conversely, patients with ID + II genotypes showed higher mean scores for negative symptoms (26.43 ± 9.46) compared to DD carriers (22.25 ± 8.68; P = 0.13), with a specific increase in passive/apathetic social withdrawal (3.71 ± 1.63 vs. 2.89 ± 1.78; P = 0.07). However, none of these differences reached statistical significance.

For general psychopathology, scores were similar between genotypes (45.37 ± 10.50 for DD vs. 43.07 ± 10.87 for ID+II; P = 0.47), as were total PANSS scores (98.69 ± 19.07 vs. 100.47 ± 22.49; P = 0.76). Additionally, no significant differences were observed between genotypes regarding smoking (OR = 0.72, 95% CI: 0.29-1.82, P = 0.49) or cannabis use (OR = 1.41, 95% CI: 0.30-6.61, P = 0.67).

## Discussion

This study aimed to investigate the association between the ACE I/D polymorphism and schizophrenia in an Eastern Algerian population. Our results showed no significant association between this variant and schizophrenia risk. Genotype and allele distributions were similar between patients and controls, and genetic models (dominant, recessive, and overdominant) confirmed the absence of statistically significant associations. Although no significant genotype-phenotype correlations were observed, a non-significant trend suggested that DD carriers exhibited higher positive symptom scores, whereas ID+II genotypes showed higher negative symptom scores.

Our findings are in line with several studies that also reported no association between the ACE I/D polymorphism and SCZ, including those conducted in Japanese [12,15], Taiwanese [13], Chinese [18], and Croatian populations [17]. However, conflicting results have been observed in other cohorts. In a Spanish population, Crescenti *et al*. [19] reported a protective role of the D allele, while in Turkish populations, the I allele appeared protective against schizophrenia [20]. An Iranian study by Mazaheri *et al*. [24], further suggested a protective effect of the I allele, but only in females. These discrepancies may be explained by genetic heterogeneity across populations [18]. Indeed, the D allele frequency in our sample (~90%) is notably higher than in Asian or European cohorts. It ranges from approximately 40% in Chinese [18] and the Japanese populations [12,15], to 50% in Spanish [19], 60% in Turkish [16,20], and 65% in Iranian cohorts [24]. This difference may reduce the power to detect genotype-based effects and underscores the importance of population-specific studies.

The biological rationale for studying the ACE gene in SCZ lies in its dual role in neurovascular regulation and dopaminergic modulation. ACE catalyzes the conversion of angiotensin I to angiotensin II and regulates dopamine turnover in the midbrain [25,26]. This interaction with dopamine is particularly pertinent to schizophrenia, since dysregulation of dopaminergic systems is central to the pathogenesis of psychotic symptoms in the disorder [27-29]. Elevated ACE levels have been found in the cerebrospinal fluid of patients with schizophrenia [10,11], while other studies reported decreased activity in specific brain regions, such as the basal ganglia in early-onset schizophrenia [30]. Moreover, experimental studies suggest that ACE regulates neuron proliferation and differentiation in the mammalian brain and influences cognition, anxiety, and memory [31-33]. These findings collectively support the hypothesis that ACE may modulate susceptibility or clinical expression of psychiatric disorders, although our study did not identify a significant genetic effect. Taken together, these biological insights provide a framework for interpreting the potential clinical implications of our findings.

While the ACE I/D polymorphism may not influence disease risk directly, our findings highlight the importance of examining genotype-symptom correlations in larger cohorts. Such relationships could ultimately contribute to more individualized approaches to symptom management in schizophrenia. This aligns with previous studies reporting associations between ACE genotypes and specific clinical profiles. Hui *et al*. [18], for example, found that this polymorphism influenced negative symptoms in Chinese patients with first-episode schizophrenia, while Nadalin *et al*. [17] observed a relationship between the D allele and psychotic features, suggesting that the I/D polymorphism may regulate ACE expression or activity, thereby influencing symptom severity. Wahlbeck *et al*. [34] also demonstrated an association between negative symptoms, such as incapacity to feel, and ACE levels in the cerebrospinal fluid of schizophrenia patients. Collectively, these findings suggest that the ACE I/D polymorphism could have subtle effects on symptomatology, particularly regarding the severity of positive and negative symptoms.

This study has some limitations, including a small sample size, which limits statistical power to detect weak genetic associations, and the focus on a single polymorphism, which does not fully represent the genetic variability of the ACE gene or its interaction with other loci. Nevertheless, it has notable strengths: rigorous genotyping methods, adjustment for age and sex, and being the first published investigation of this association in an Algerian population. It thus provides novel data from North Africa and contributes to the global effort to understand the genetic architecture of schizophrenia across diverse populations.

## Conclusion

This study did not find a significant association between the ACE I/D polymorphism and SCZ in an Eastern Algerian population. Genotype and allele frequencies, as well as analyses under different genetic models, showed no significant differences between patients and controls. Although not statistically significant, symptom trends were noted, with DD genotypes showing higher positive symptoms and ID+II genotypes showing higher negative symptoms. These findings are consistent with previous studies in other populations. As the first published investigation of this polymorphism in an Algerian cohort, our results provide novel data from North Africa and underscore the importance of conducting population-specific studies to better understand the genetic architecture of SCZ.

### What is known about this topic

- Schizophrenia is a multifactorial psychiatric disorder influenced by genetic and environmental factors;
- The ACE I/D polymorphism affects enzyme activity, dopamine regulation, and neuroinflammation;
- Prior studies on ACE I/D polymorphism and schizophrenia showed inconsistent results across populations.

### What this study adds

- First investigation of ACE I/D polymorphism and schizophrenia in an Eastern Algerian population;
- No significant association detected between ACE I/D polymorphism and schizophrenia risk;
- Observed non-significant genotype-related trends in positive and negative symptom severity.
